# How to improve the efficiency and the safety of real-time ultrasound-guided central venous catheterization in 2023: a narrative review

**DOI:** 10.1186/s13613-023-01141-w

**Published:** 2023-05-25

**Authors:** Nicolas Boulet, Laurent Muller, Claire M Rickard, Jean-Yves Lefrant, Claire Roger

**Affiliations:** 1 Department of Anesthesiology, Critical Care, Intensive Care, Pain and Emergency Medicine, Nîmes University Hospital, Place du Professeur Debré, Gard, 30900 Nîmes, France; 2 IMAGINE, UR-UM 103, University of Montpellier, Nîmes University Hospital, Nîmes, France; 3 School of Nursing, Midwifery, and Social Work & Herston Infectious Diseases Institute, The University of Queensland & Royal Brisbane and Women’s Hospital, Brisbane, QLD Australia; 4 Alliance for Vascular Access Teaching and Research, Menzies Health Institute Queensland, Griffith University, Brisbane, QLD Australia

**Keywords:** Central venous catheterization, Ultrasound, Catheter-related complications, Subclavian vein

## Abstract

Central venous catheterization (CVC) is a frequent procedure, practiced by intensivists, anesthesiologists and advanced practice nurses in intensive care units and operative rooms. To reduce CVC-associated morbidity, it is essential to strive for best practices, based on the latest evidence. This narrative review aims to synthesize current knowledge on evidence-based best practices for CVC that improve the use and feasibility of real-time ultrasound-guided insertion procedures. Optimization of the vein puncture technique and the development of new technologies are discussed to reinforce the use of the subclavian vein catheterization as first choice. The search for alternative site of insertions, without increasing infectious and thrombotic risks, deserves further research.

**Supplementary Information:**

The online version contains supplementary material available at 10.1186/s13613-023-01141-w.

## Introduction

Central venous catheterization (CVC) is indicated in nearly 75% of intensive care patients [1], allowing the administration of venotoxic drugs or vasopressors, vascular filling, parenteral nutrition, repeated blood sampling, and hemodynamic monitoring by central venous pressure measurement. The main sites for CVC insertion are the internal jugular vein (IJV), the subclavian (SV) or proximal axillary vein (AV), and the femoral vein (FV). Although CVC is indicated to improve the management of intensive care patients, it is associated with complications that could be separated in two types, according to the timing of the venipuncture:

- Immediate complications (8–15%), such as pneumothorax, arterial puncture, bleeding complications, cardiac arrhythmias, and catheter malposition [2–5]. It has been largely reported that some patient (BMI < 20 kg m^−2^) and operator-related (male gender, limited experience, and ≥ 2 skin punctures) risk factors are associated with a higher rate of immediate complications [5].
- Late complications (2%), corresponding mainly to catheter-related infections (CRI) and catheter thrombosis, and dislodgement [6]. They can lead to an increase in hospital length of stay, hospitalization costs and mortality in intensive care patients [7].

This narrative review aims to synthesize current evidence-based best practices for CVC use, and to highlight ways to improve the use and feasibility of real-time ultrasound (US)-guided puncture. We will therefore detail new developments that will provide areas for research and improvement of CVC outcomes by firstly summarizing the latest international guideline recommendations in critical care (2020) [1, 8, 9], and then by focusing on published innovations since this date. All in-ICU or operating room studies regarding US-guided CVC with short-term catheters in adult patients since 2020 were screened and reviewed from the Pubmed database (Additional file 1). Earlier studies were extracted from international guidelines.

## What is already recommended: a brief reminder

### Real-time US-guidance

US-guidance improves the efficiency, safety, and comfort during CVC [1, 8, 10]. To reduce the incidence of immediate complications, it is recommended to use the real-time US-guidance for CVC as the first-line procedure in all puncture sites [1, 8–10]. The superiority of real-time US-guidance (or “direct” US-guidance) has been demonstrated over indirect US-guidance (corresponding to a preprocedural US localization of the vessels, followed by a blind puncture) [2, 3]. The US-guidance reduces the immediate complications in a threefold manner for the jugular and femoral sites, and twofold for the subclavian/proximal AV site [2–4]. However, US-guidance remains underused, employed in 36–68% of insertions (less than 30% of cases for the subclavian site) [6, 11, 12]. The main reasons were: usefulness (36%), unavailability of the US machine (33%), or a prolonged perceived procedure time (19%) [12].

Although real-time US-guided CVC insertion has an effective reduction of immediate complications and improvement of comfort, its effect on the number of infectious complications remains debated, because of conflicting data [13–15]. Surprisingly, a post hoc analysis of three large randomized controlled trials (RCTs) [16] found a potentially increased infectious risk with real-time US-guidance, perhaps due to an increased risk of asepsis errors with the equipment. It is important to remind that regardless of the use of real-time US-guided insertion, CVC procedures must always be performed under strict aseptic conditions (cap, mask, sterile gown, sterile gloves, large sterile fields), with maximum hand hygiene [1, 9].

### Traditional puncture sites

The infraclavicular exit site (proximal AV/SV) is recommended in first intention, in order to reduce late complications [1], as it has been shown to be the exit site with the lowest infectious and thrombotic complications [6, 17–19]. However, the infraclavicular site remains rarely used (< 30% of a regular users), because of the perception of a higher procedural difficulty by the operators, and the risk of pneumothorax [11, 12].

The other traditional insertion sites (IJV, FV) should only be used in case of contraindication to the infraclavicular AV/SV catheterization (respiratory instability, thrombosed vein, coagulation disorders, …) [1], or when those sites have been exhausted in the case of repeated catheters. International recommendations do not support the use of the IJV approach over the FV approach in order to reduce infectious complications [1]. However, several studies seem to support the IJV catheterization over the FV site, with a reduction in catheter colonization [6, 20] or CRI [21–23]. A superiority of the IJV approach is suggested in patients with a high body mass index (> 28.4 kg m^−2^) [24, 25] or for an insertion time greater than 5 days [26]. In addition, this site is more difficult to maintain and monitor for nurses, being close to the perianal area and obscured by bed linen. This should prompt the greatest caution concerning the FV catheterization. The FV approach is also at high risk of thrombosis [6]. These data have led several "bundle of catheter care" to avoid the FV catheterization as long as possible [27]. Our opinion is to use the FV site only if AV/SV catheterization, then IJV (superior vena cava territory thrombosis, local surgery, etc.), is not possible.

### Periprocedural US analysis

A preprocedural US evaluation of all puncture sites is recommended for the detection of a local disease or abnormal anatomy, and to select the best catheterization site option [8, 9]. The RaCeVA protocol has been previously described for this purpose [28, 29]. This protocol is an easy, rapid, and systematic assessment of the six central veins that can be theoretically punctured with US-guidance in the supraclavicular and infraclavicular areas. However, this procedure has not been compared with standard of care.

An immediate postprocedural vascular, cardiac, pleural and lung US analysis is recommended [8, 9]:

- To detect and prevent catheter malposition, as it has been shown to be well tolerated, feasible, quickly performed, and more accurate, economical and faster than a chest radiograph [8].
- To rule out potential pleural-pulmonary complications (mainly pneumothorax), as it has been shown to be feasible, more sensitive and less costly than a chest radiography, which allows a quicker diagnosis at the bedside of respiratory complications, and avoids radiation exposure [8].

This procedure may be enhanced by bubble test that improves visualization of the catheter tip [30].

Despite these advantages, self-reported use of postprocedural US and discontinuation of routine chest radiography is low [31, 32].

### Some areas for improvement

The current challenge is to facilitate and encourage compliance with the guidelines and to develop new procedures or technologies to further improve the efficiency, safety, and practitioners’ comfort of real-time US-guided CVC. The best puncture technique remains debated in the current scientific literature and will be discussed in this review. Furthermore, an improvement of learning techniques through the development of specific training programs including simulation is recommended [1, 8, 33–35]. Then, the implementation of new technologies allowing the improvement of comfort and safety of venous puncture, could facilitate the use of periprocedural US analysis, and help to rehabilitate the use of the subclavian site to decrease late catheter-related complications. Finally, as an alternative to a potentially difficult AV/SV catheterization, it may also be wise to look for alternative locations for CVC, with a comparable risk of infectious and thrombotic complications.

## Optimal choice of the puncture technique

**Fig. 1:**
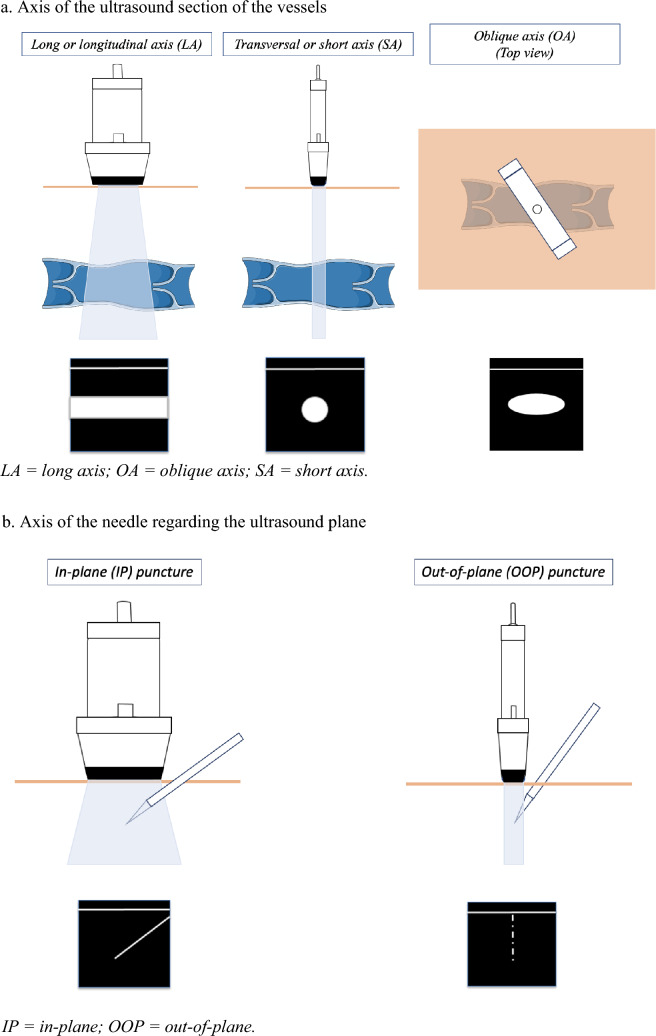
Different axes in ultrasound-guided venous puncture

Because confusions persist in the scientific literature, it is important to remember that there are two different axes in real-time US-guided CVC (Fig. 1):

- The axis of the vessel (Fig. 1a): this corresponds to the US view of the vessel. Depending on the section of the vessel crossed by the US beam, it will appear longitudinal [long axis (LA)], transverse [short axis (SA)], or oblique [oblique axis (OA)] [8].
- The axis of the needle (Fig. 1b): this corresponds to the axis of the needle in relation to the US beam. If the needle is introduced longitudinally to the US probe and beam, the puncture is defined as "in-plane" (IP), and the needle appears in its entirety on the US screen. On the contrary, if the needle is introduced perpendicularly to the US beam, in the center of the probe, the puncture is defined as "out-of-plane" (OOP), and the needle appears as a hyperechogenic point progressing on the US screen [8].

In each puncture site, there is still a debate about the superiority of IP versus OOP puncture.

The puncture of the posterior wall of the vein is a frequent intermediate outcome observed in simulation trials. Although never demonstrated, this criterion could reflect the risk of immediate complications visualized in clinical practice. In these studies, we observe a reduction in puncture of the posterior wall of the vein associated with the IP versus the OOP technique [36–42], probably due to a complete visualization of the needle during IP puncture.

From a clinical perspective, one RCT about proximal AV/SV catheterization found a significant superiority of the OOP-SA versus IP-LA puncture with a shorter insertion time (69 versus 98 s, *p* = 0.040), a higher overall success rate (96 vs. 78%, *p* < 0.001), first-puncture success rate (86 vs. 67%, *p* = 0.003), and first-puncture single-pass success rate (72 vs. 48%, *p* = 0.002), with fewer needle redirections (0.39 ± 0.88 vs. 0.88 ± 1.15, *p* = 0.001), skin punctures (1.12 ± 0.38 vs. 1.28 ± 0.54, *p* = 0.019), and complications (3 vs. 13%, *p* = 0.028, with arterial puncture = 7 versus 0%, *p* = 0.014) [43]. These results are inconsistent with a retrospective study that found a greater one-attempt success rate with the IP-LA technique, but with a longer procedure duration, in experienced operators [44]. The two groups were comparable with complications and clinical characteristics. In the IJV site, several meta-analyses of RCTs have been published on this subject, which do not find superiority of one type of puncture of the IJV [45–48]. Indeed, since this procedure is relatively simple to perform and the vessel is superficial, it seems difficult to show a difference between the different types of punctures on the effectiveness or complications of the procedure.

Given the rarity of the incidence of serious immediate complications during CVC, current data do not allow to conclude with certainty to the superiority of one technique over another in terms of complications. There is still a need for scientific evidence based on large RCTs. It is also difficult to conclude given the disparity of training and expertise in the various techniques among the teams conducting these studies.

From our point of view, the OOP puncture is simpler to learn initially, but ultimately more complex to perform safely. Indeed, to obtain a safe and quality puncture, it requires a complex dynamic follow-up of the needle tip. The needle trajectory should be anticipated, which can be facilitated by mental visualization of an isosceles triangle, between the vessel, the needle, and the skin [49]. On the opposite, the IP puncture, which is more technical to learn, allows then a follow-up of the needle on all its length, with a safe visual control of the needle during all the procedure. There is a lack of scientific data to support this statement.

As alternatives, the IP-SA or IP-OA punctures could be used. These news techniques could allow a panoramic view of arteries and nerves, to avoid inadvertent damage to these structures, combined with a visualization of the entire needle during the procedure [50]. In a retrospective cohort [51], the IP-OA puncture of the AV shows its feasibility (96% of first-attempt success) and safety (no arterial puncture or pneumothorax, and only 2.5% of guide wire malposition). One limitation is that a single expert operator in US-guided CVC performed all the procedures. Further studies are needed to determine the usefulness of these new approaches. More recently, 3 RCTs in experienced operators [37, 52, 53] found an equivalence of the IP-OA puncture versus the OOP-SA view, in terms of efficiency and security. A prospective observational study found a higher incidence of complications in the OOP-SA group (56.7% vs 16.7%) in experienced operators [38], with no significant interoperator variability in terms of acute complications and success rate. Although the results are contradictory, it appears that IP-OA puncture seems to be a promising alternative in terms of efficacy and safety.

## New technologies

As CVC can be difficult in certain deep sites (as subclavian) or in certain patients (obesity, previous local surgery, etc.), new real-time US-guidance technologies have been developed for improving the comfort and safety of this procedure.

### Magnetic devices

Magnetic devices improve the real-time visualization of the needle and its location relative to the US beam, which could theoretically lead to a better success rate and lower complication rate. A recent randomized controlled simulation study assessed the efficacity and the safety of the AV/SV catheterization with a new magnetic needle-pilot system versus a usual US-guidance [54]. The use of the needle-pilot device was associated with a significant shorter time to successful cannulation, fewer number of skin punctures, fewer posterior wall punctures of the vein, fewer needle redirections, and a better operator comfort during puncture. These differences were reported regardless of the operator experience. These promising findings obtained on a simulation mannequin should be confirmed by an RCT in intensive care patients.

### Specific needles

New photoacoustic needles are others promising technologies using the following principles [55]: an optical fiber is inserted, without occluding, into the lumen of a standard needle, transmitting a pulsed laser light from an external laser source, then translating into US waves, which are received by the US probe for imaging. A pilot study founding a significant improved ability to identify the needle tip on recorded videos [55]. Further studies are needed to assess this promising technology in clinical practices.

In addition, Arya et al. [56] devised a guard which can be slid and fixed over the CVC needle at a desired length (measured through US) preventing the needle from penetrating deeper into the skin beyond this guard. This RCT on 419 patients found a significantly higher successful IJV cannulation in the first attempt with the guard (98.6 vs. 85.7%, *p* = 0.007), with less complications, such as posterior wall puncture (0.5 vs 8.61%, *p* = 0.001) and common carotid puncture (0 vs 7.18%, *p* = 0.001).

### Smart glasses

Smart glasses are also another type of emerging technology to assist in performing US-guided procedures. Different models exist, but these are smart glasses allowing the visualization of the US screen in augmented reality. This could allow a better synchronization between the axis of the eyes, the needle, and the US screen. A clinical study investigated this technique in pediatric patients requiring US-guided radial arterial catheterization [57]. No differences has been reported in procedural time, in the number of skin punctures or needle restrictions between groups. A simulation study on a phantom model found an improved procedure time of 17% in novices, but an increased duration by 5% in experienced participants, which may reflect the experts' training and experience bias [58]. This technique has not been assessed for CVC in intensive care patients.

### 3D-biplane technologies

A 3D-biplane probe could theoretically allow simultaneous LA and SA visualization of the vessel, and thus improve the efficiency and safety of CVC. However, the literature seems contradictory. A non-randomized prospective study found an increased efficiency, with an improved success rate at the first-attempt success rate (90 vs 50%, *p* < 0;0001) with this technique [59]. However, the first-attempt success rate was surprisingly low in the control group. These favorable results were not confirmed by recent RCTs in simulated or real IJV catheterization [60, 61], probably due to a higher mental effort of this new technique.

## Development of new access sites (Fig. 2, Table 1)

**Fig. 2:**
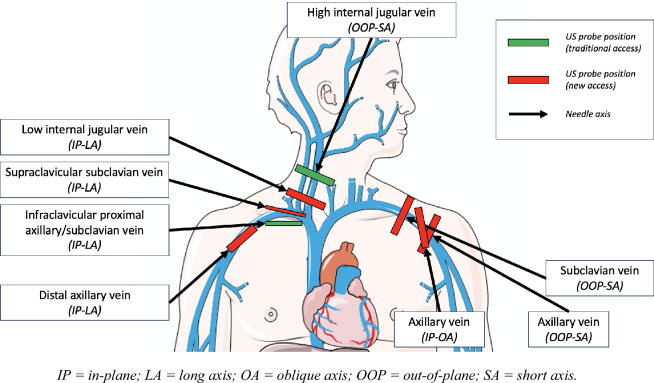
Development of new access sites

**Table 1:** Summary of new access sites and puncture techniques data

**Low internal jugular vein puncture**
Equivalent efficiency and immediate complications between the IP-LA and the OOP-SA techniques. Perhaps a slightly better efficiency and safety with the IP-OA puncture
About the infectious data of the low IJV approach (an alternative to the classical IJV access), there is a very low level of evidence (mainly based on one observational study). Reduction of CRBSI compared to the conventional IJV puncture, but limited evidence about the equivalence of CRBSI compared to SV approach.
**Supraclavicular subclavian or brachiocephalic vein puncture (IP-LA)**
Non-inferior or even superior efficiency compared to the infraclavicular proximal AV/SV puncture and to the OOP-SA IJV puncture, depending on the studies reviewed.
Less catheter misplacements than the infraclavicular proximal AV/SV catheterization.
Equivalent immediate complications compared to the infraclavicular SV and the OOP-IJV access sites.
Infectious complications not or poorly documented (a small study with poor quality data collection). Further studies are needed to compare catheter-related infections with infraclavicular proximal AV/SV catheterization.
**Distal axillary vein puncture**
Non-inferior efficiency compared to the proximal AV/SV puncture, but maybe more difficult puncture
Maybe more immediate complications than the proximal AV/SV puncture (16.1 versus 6.6%, including 6.5 versus 0% of arterial punctures, non-statistically significant), but with less pneumothorax (3.3% versus 0%, non-statistically significant).
The IP-LA technique is more used, but the IP-OA and IP-SA punctures could be good alternatives (further studies needed).
Further studies needed to assess infectious complications

*AV* axillary vein, *IJV* internal jugular vein, *IP* in-plane, *LA* long axis, *OA* oblique axis, *OOP* out-of-plane, *SA* short axis, *SV* subclavian vein

New puncture sites are being studied, with the aim of approaching the level of infectious risk of the subclavian venous approach, without increasing the immediate mechanical complications. The challenge is to catheterize the central vein as far as possible from the contaminated sites (Ear, Nose, and Throat (ENT) sphere and armpit).

### Distal internal jugular site

The distal (or low or posterior) IJV approach has been previously described and studied in several trials [62–66]. A single-center observational study found a lower incidence of CRBSI compared to the high (or central) IJV access (1.2 versus 4.8 per 1000 catheter-day, OR = 3.9; 95% confidence interval (CI) 1.1–infinite; *p* = 0.03) [63]. The same study was performed comparing the low IJV and the SV approaches [62], and did not find any differences in the incidence of CVC-RB. More recently, our team has published a prospective randomized controlled open-label trial comparing low IJV and proximal AV/SV catheterization [66]. This study on 201 patients found an overall success rates for IJV and proximal AV/SV sites of 96% and 89%, respectively. First puncture success rates were 90% and 80%, respectively. The median overall procedure duration from US preprocedural screening to guidewire insertion was 8 and 10 min, respectively. Overall immediate complication rates for IJV and proximal AV/SV sites were 11.6% and 14.6%, respectively. This trial also found a comparable risk of catheter thrombosis (0% in each group) and catheter colonization (6.8% in proximal AV/SV approach and 7.9% in low IJV approach). CRI seemed to be slightly higher in the low IJV group (2.6% versus 0%). However, it was a pilot study, with an objective of estimation (not comparison). This trial was not powered to show a difference between the groups.

### Supraclavicular subclavian and brachiocephalic sites

Other recent approaches, in the supraclavicular area (IP-LA subclavian or brachiocephalic vein) have also been described [67–73]. In comparison with the IJV [69, 70] or the infraclavicular SV catheterization [67, 71], these techniques seem to be as safe (or even safer) in terms of immediate complications.

In comparison with the OOP-SA IJV puncture, the IP-LA supraclavicular subclavian vein (SSV) catheterization shows a significantly higher first-attempt success rate (83.2% vs 63.2%, *p* = 0.001), a shorter insertion time (43.98 ± 26.77 vs. 53.12 ± 40.21 s; *p* = 0.038), a fewer number of puncture attempts (1.16 ± 0.39 vs. 1.47 ± 0.71; *p* < 0.001), a fewer number of needle redirections (0.69 ± 0.58 vs. 1.17 ± 0.95; *p* < 0.001), less difficulties in guidewire advancement (2.4% vs. 27.4%; *p* < 0.001), and less venous collapse (2.4%, vs. 18.4%; *p* < 0.001) in a recent RCT [69]. A limitation of this study is the success rate at the first internal jugular catheterization puncture, which appears low compared with other data in the literature. No difference was found between this two methods in another recent RCT [70]. About the pure puncture technique, in the supraclavicular area, the OOP approach seems to be difficult to use for anatomical reasons (interposition of the clavicle).

In comparison with the IP-LA infraclavicular proximal AV/SV, a RCT reported a significantly lower total procedural time, time for visualization, puncture, and catheterization with the IP-LA supraclavicular subclavian catheterization [67]. No statistical difference was found in total and first-attempt success rates. Another recent RCT found an improvement in the composite outcome of immediate complications with the IP supraclavicular puncture, mainly related to a decrease in the number of catheters misplacement [71].

In case of similar infectious rate, these supraclavicular approaches could represent interesting alternatives to the classically used approaches because of their easy execution and their lower risk of immediate complications in most studies cited. Only one study reported a higher infection rate in the SSV approach group, but not statistically significant [67]. This is a small RCT on 110 patients. It should be noted that no percentages are reported in the manuscript. High-level evidence studies are needed to assess the infectious complications of these techniques.

### Distal axillary site

The location of the puncture site (distal AV or proximal AV/SV) seems comparable in terms of feasibility and safety. One RCT found a non-inferiority between the IP-LA proximal AV/SV and the distal AV catheterization in terms of efficacy and immediate complications [74]. However, the distal AV approach resulted in more arterial punctures (6.5% vs 0%), but less pneumothorax (0% vs 3.3%). Another RCT in cardiac surgery found that the IP-LA proximal group had a higher first-puncture success rate (75.8% vs. 51.5%, *p* < 0.001), fewer average number of attempts (1.3 ± 0.7 vs 1.7 ± 0.9, *p* < 0.01), less access time (20 [15; 28] versus 30 [19; 42] s, *p* < 0.001), and less successful cannulation time (123 [112; 136] versus 142 [133; 156], *p* < 0.001) than the IP-LA distal group [75]. The rate of complications was similar in the two groups. These last data were confirmed in another RCT on elderly patients (one attempt and overall success rates were significantly higher in the proximal axillary vein group, compared with the distal axillary vein group (71.4% vs 42.0%, *p* = 0.003; 79.6% vs 54.0%, *p* = 0.007)) [76]. Further studies on larger populations are needed to assess the safety equivalence of these two puncture sites of the AV. Concerning infectious complications, only one retrospective study on IP-OA of distal AV catheterization reported only 0.0365 CRBSI per 1000 catheter days [51]. Further studies need to compare CRI between distal and proximal approach of the AV catheterization.

## Conclusion

After reminding and encouraging the respect of the international guidelines, this narrative review discussed new developments in improving CVC in the intensive care units. The rehabilitation of the US-guided subclavian puncture is an important issue to reduce infectious and thrombotic complications of CVC. This could be achieved by improving the safety and operator’s comfort of CVC insertion, with the help of new technologies and optimal puncture techniques. Numerous access sites are developing as alternatives to traditional sites (proximal AV/SV and IJV approaches). However, there are few studies comparing the infectious complications of these different sites. The search for an equivalence of late complications on potentially more easily accessible puncture sites could be an important issue for the future. However, since it is not yet possible to conclude with certainty that one technique is superior to the other, clinicians should know all techniques to personalize the CVC to each patient’s characteristics.

### Supplementary Information

**Additional file 1: **Search strategy

## Data Availability

Not relevant.
