# Correction: How to improve the efficiency and the safety of real-time ultrasound-guided central venous catheterization in 2023: a narrative review

**DOI:** 10.1186/s13613-023-01212-y

**Published:** 2023-11-25

**Authors:** Nicolas Boulet, Laurent Muller, Claire M. Rickard, Jean-Yves Lefrant, Claire Roger

**Affiliations:** 1 Department of Anesthesiology, Critical Care, Intensive Care, Pain and Emergency Medicine, Nîmes University Hospital, Place du Professeur Debré, Gard, 30900 Nîmes, France; 2 IMAGINE, UR-UM 103, University of Montpellier, Nîmes University Hospital, Nîmes, France; 3 School of Nursing, Midwifery, and Social Work & Herston Infectious Diseases Institute, The University of Queensland & Royal Brisbane and Women’s Hospital, Brisbane, QLD Australia; 4 Alliance for Vascular Access Teaching and Research, Menzies Health Institute Queensland, Griffith University, Brisbane, QLD Australia

**Correction: Annals of Intensive Care (2023) 13:46** 10.1186/s13613-023-01141-w

In the original publication of the article, the author names were abbreviated as N. Boulet, L. Muller, C. M. Rickard, J. Y. Lefrant and C. Roger. The corrected author names should read as Nicolas Boulet, Laurent Muller, Claire M Rickard, Jean-Yves Lefrant, Claire Roger. The original article [1] has been corrected.
