# Supplementary material for: How to improve the efficiency and the safety of real-time ultrasound-guided central venous catheterization in 2023: a narrative review
Source: Ann Intensive Care. 2023 May 25;13:46. doi: 10.1186/s13613-023-01141-w (PMC10212873; doi:10.1186/s13613-023-01141-w)
Supplement: Supplementary file 1 — Additional file 1: Search strategy [file 13613_2023_1141_MOESM1_ESM.docx]

**Additional file 1: search strategy**

(("catheterization, central venous"[MeSH Terms] OR "central venous pressure"[MeSH Terms] OR "central venous catheters"[MeSH Terms] OR "central venous catheter*"[All Fields] OR "central venous pressure"[All Fields] OR "central venous line*"[All Fields] OR "central line insertion*"[All Fields] OR (("lung"[MeSH Terms] OR "lung"[All Fields] OR "pulmonary"[All Fields]) AND "arter*"[All Fields] AND "flotation*"[All Fields]) OR (("veins"[MeSH Terms] OR "veins"[All Fields] OR "venous"[All Fields] OR "vein*"[All Fields]) AND ("cannulate"[All Fields] OR "cannulated"[All Fields] OR "cannulating"[All Fields] OR "cannulator"[All Fields] OR "cannulators"[All Fields] OR "cannulisation"[All Fields] OR "cannulization"[All Fields] OR "cannulized"[All Fields] OR "catheterization"[MeSH Terms] OR "catheterization"[All Fields] OR "cannulation"[All Fields] OR "cannulations"[All Fields] OR ("access"[All Fields] OR "accessed"[All Fields] OR "accesses"[All Fields] OR "accessibilities"[All Fields] OR "accessibility"[All Fields] OR "accessible"[All Fields] OR "accessing"[All Fields]) OR "catheter*"[All Fields]))) AND ("ultrasonics"[MeSH Terms] OR "ultrasonography"[MeSH Terms] OR "ultrasonography, doppler"[MeSH Terms] OR "echocardiography, doppler"[MeSH Terms] OR "ultrasonography, doppler, color"[MeSH Terms] OR "ultraso*"[All Fields] OR "Doppler"[All Fields] OR "echograph*"[All Fields]) NOT ("adolescent"[MeSH Terms] OR "birth cohort"[MeSH Terms] OR "child"[MeSH Terms] OR "infant"[MeSH Terms] OR "adolescent"[Title/Abstract] OR "birth cohort"[Title/Abstract] OR "child"[Title/Abstract] OR "infant"[Title/Abstract] OR "pediatric*"[Title/Abstract])) NOT ("animals"[MeSH Terms] OR "animals"[Title/Abstract])
